# Bio-Based Thermo-Reversible Aliphatic Polycarbonate Network

**DOI:** 10.3390/molecules25010074

**Published:** 2019-12-24

**Authors:** Pierre-Luc Durand, Etienne Grau, Henri Cramail

**Affiliations:** CNRS, University Bordeaux, Bordeaux INP, LCPO, UMR 5629, F-33600 Pessac, France; durand.pierreluc@gmail.com (P.-L.D.); egrau@enscbp.fr (E.G.)

**Keywords:** polycarbonates, furan-maleimide, Diels-Alder, bio-based, fatty acids

## Abstract

Aliphatic polycarbonates represent an important class of materials with notable applications in the biomedical field. In this work, low Tg furan-functionalized bio-based aliphatic polycarbonates were cross-linked thanks to the Diels–Alder (DA) reaction with a bis-maleimide as the cross-linking agent. The thermo-reversible DA reaction allowed for the preparation of reversible cross-linked polycarbonate materials with tuneable properties as a function of the pendent furan content that was grafted on the polycarbonate backbone. The possibility to decrosslink the network around 70 °C could be an advantage for biomedical applications, despite the rather poor thermal stability of the furan-functionalized cross-linked polycarbonates.

## 1. Introduction

Aliphatic polycarbonates (APC) raise considerable interest in the last decades for their specific characteristics i.e., biocompatibility and biodegradability [1]. Different routes can achive their synthesis, but these polymers are mainly synthesized by ring-opening polymerization (ROP) of cyclic carbonate monomers allowing for a good control over the polymer’s microstructure [2]. The advantage of designing functional polycarbonates when compared to traditional polytrimethylenecarbonate (PTMC) lies in the modulation of their physico-chemical properties for specific needs, thus broadening and improving their performance characteristics. Functional polycarbonates can be synthesized by direct polymerization of a functional monomer or by post-polymerization chemical modification [3]. Besides, the use of APC from renewable resources, such as vegetable oils, is very interesting due to environmental concerns and also to seek new functionalities [4].

In addition, polycarbonate networks with elastomeric properties are desirable for a large number of biomedical applications, particularly in the emerging field of soft tissue engineering or drug delivery [5,6,7,8,9,10]. Thus, several research groups have studied various cross-linking methods for obtaining polycarbonate materials [11,12,13,14,15,16]. However, the main disadvantage of these cross-linked materials is their inability to be reformed or recycled. As a result, the development of self-healing polymers has been a very active area of research over the last decade [17,18,19,20,21]. In 2013, the World Economic Forum even named self-healing materials among the top 10 emerging technologies and could be of interest in many areas, such as protective coatings, biomedical applications, piping, and electronics [18,22]. Various stimuli can be employed to activate the reversibility of the transformation. Nevertheless, temperature and light are the two stimuli, which are most commonly used in the synthesis of “stimuli-responsive” self-healing materials.

A thermal process is convenient and effective for the treatment of materials and it can be easily implemented to any object. As a result, heat-induced reversible reactions are attractive for building up thermal-responsive self-healing materials. The most studied thermally reversible reactions are based on the Diels–Alder (DA) chemistry. The latter has been widely used in macromolecular chemistry and mostly based on the furan/maleimide chemistry [23,24,25,26,27,28,29,30,31,32].

Therefore, the purpose of this investigation is to synthesize new, thermally reversible, polycarbonate networks via Diels–Alder and retro-Diels–Alder reactions. The general method is based on the synthesis of low Tg polymers containing dangling furan groups, followed by their Diels–Alder cross-linking with an appropriate bis-maleimide cross-linking agent.

First, the grafting of pendent furan moieties will be detailed on a fatty acid based polycarbonate. Subsequently, the thermal reversibility of the cross-linking will be demonstrated. Finally, the influence of the cross-linking density on the polycarbonate properties will be studied.

## 2. Results and Discussion

### 2.1. Grafting of Furan Moieties on the Polymer

Aliphatic polycarbonate P(**NH-Und-6CC**) was used as the starting materials, since its synthesis by ROP of **NH-Und-6CC** is well-controlled and it can be performed on a multi-gram scale according o already published protocols (see Supplementary Materials for the full protocols) [16,21].

Therefore, the first task was the preparation of a furan-functionalized polycarbonate. Thiol-ene click chemistry appears to be straightforward to graft a desired functionality onto the polymer backbone, thanks to the available terminal unsaturation on each monomer unit.

**Scheme 1:**
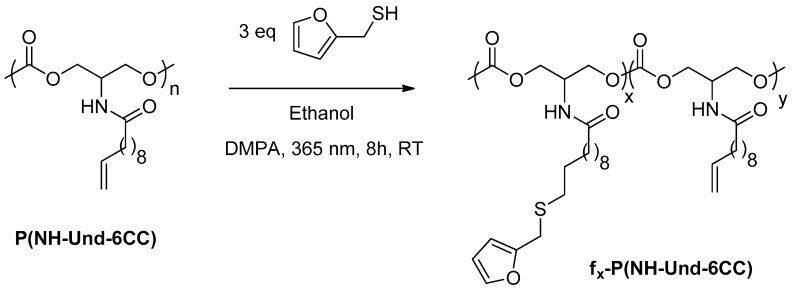
Experimental procedure for the preparation of furan-functionalized P(NH-Und-6CC).

The thiol-ene reaction between P(**NH-Und-6CC**) and the commercially available furfuryl mercaptan was performed at room temperature in ethanol at 2 mol L^−1^ for 8 h under UV irradiation (365 nm) (Scheme 1). DMPA was used as a radical initiator. The mixture was irradiated through an optical fiber (365 nm) to improve the conversion and decrease the reaction time. Additionally, three equivalents of furfuryl mercaptan with respect to C=C double bond were added for the same reasons. The polymer was recovered by precipitation in cold methanol.

^1^H NMR spectroscopy followed the thiol-ene reaction for 8 h (Figure S1). The disappearance of the characteristic signals of the terminal double bond (5.0 and 5.8 ppm) and the appearance at 2.50 ppm of the proton in α position of the sulfur atom (towards the polymer chain) enable the monitoring of the reaction. The signal at 2.25 ppm (integrated for 2) of the protons in α position of carbonyl from the amide group was taken as an internal reference to determine the furan content in the copolymer, thanks to the ratio of the peak at 2.25 and 2.50 ppm.

**Figure 1:**
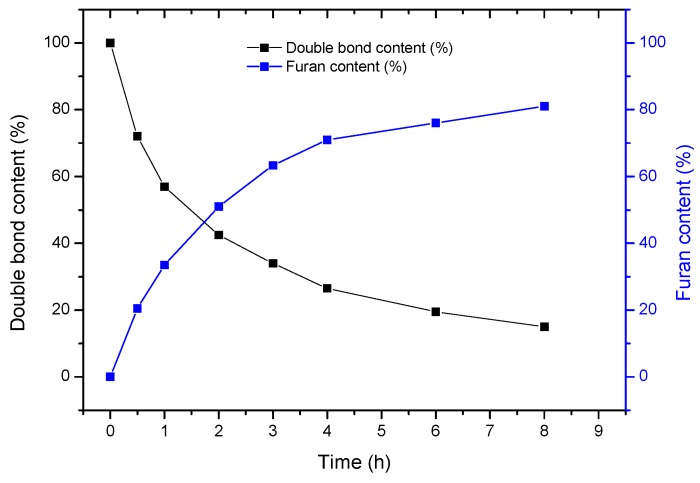
Kinetic profile of the P(NH-Und-6CC) functionalization by furan moieties.

The evolution of the furan and the double bond content in the polycarbonate was plotted as a function of time (see Figure 1). The polymer displayed a moderate reactivity towards furfuryl mercaptan, since only 85 mol% of furan was grafted on the polycarbonate backbone after 8 h of reaction. However, the amount of furan that is grafted on the polycarbonate backbone can easily be adjusted by varying the reaction time. In addition, one can assume that the higher the furan content, the higher the cross-linking density. Thus, this control of the furan content is interesting to investigate the influence of the cross-linking density on the network properties.

**Figure 2:**
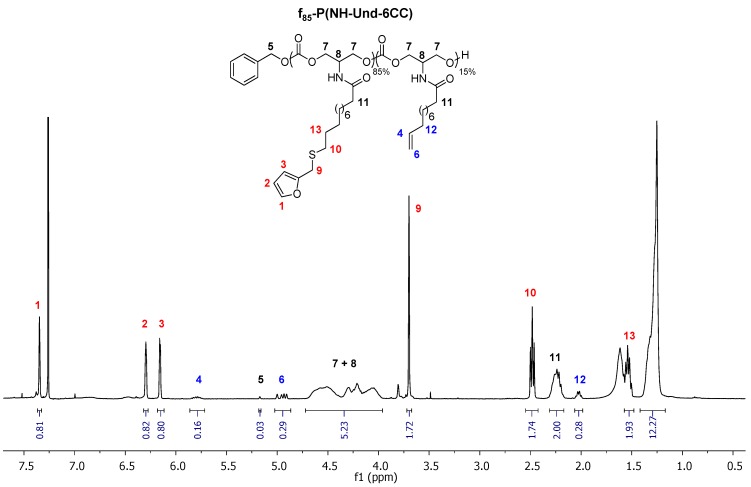
^1^H NMR of f_85_-P(NH-Und-6CC) in CDCl_3._

^1^H NMR characterized the resulting 85 mol% furan-functionalized polycarbonate, f_85_-P(**NH-Und-6CC**) (see Figure 2). All of the peaks were assigned confirming the structure of the furan-functionalized polymer.

Various polycarbonates were synthesized with different targeted furan contents while using the kinetic master curve. Table 1 summarizes the results.

First, it is noteworthy that the furan content is in a good agreement with respect to the kinetic profile (1). T_g_s of these polycarbonates (Figure 1) range from 23.1 to −59.6 °C as a function of the furan content increase. Such a T_g_ decrease can be explained by the flexibility that the sulfur atoms introduced to the polymer chains. In addition, the SEC traces (Figure S2) indicated that the polymer molar masses increase is proportional to the furan content and testified no apparent degradation of the polymer during the grafting reaction.

Therefore, several pendent furan-containing polycarbonates have been successfully synthesized. The reversible cross-linking will now be investigated thanks to the introduction of a bis-maleimide as cross-linking agent.

### 2.2. Reversible Cross-Linking and Reprocessability

**Scheme 2:**
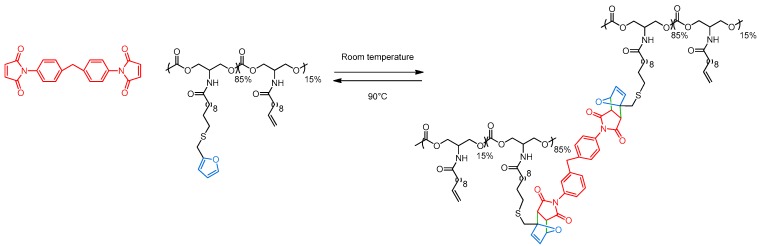
Cross-linking reaction between f_85_-P(**NH-Und-6CC**) and bis-maleimide.

The thermal reversibility of the cross-linking reaction was first demonstrated while taking f_85_-P(**NH-Und-6CC**) as starting furan-containing polymer. Scheme 2 summarizes the mechanism that is involved in the cross-linking reaction.

The cross-linked polycarbonate material was obtained, as follows. The polymer f_85_-P(**NH-Und-6CC**) was dissolved in chloroform (CHCl_3_) at 1 g mL^−1^ in a vial. The bis-maleimide cross-linking agent was then added to the previous solution (0.5 equiv., with respect to furan groups) and the mixture was homogenized by vortex stirring until a clear yellow solution appears (the color is due to the bis-maleimide molecule). The vial was closed tightly and then heated up to 60 °C for 10 min.

The warm solution was poured in a Teflon mold and the chloroform was allowed to gently evaporate overnight at room temperature. A cross-linked film was obtained (Diels–Alder reaction) and was dried under reduced pressure for several hours to remove the traces of chloroform.

**Figure 3:**
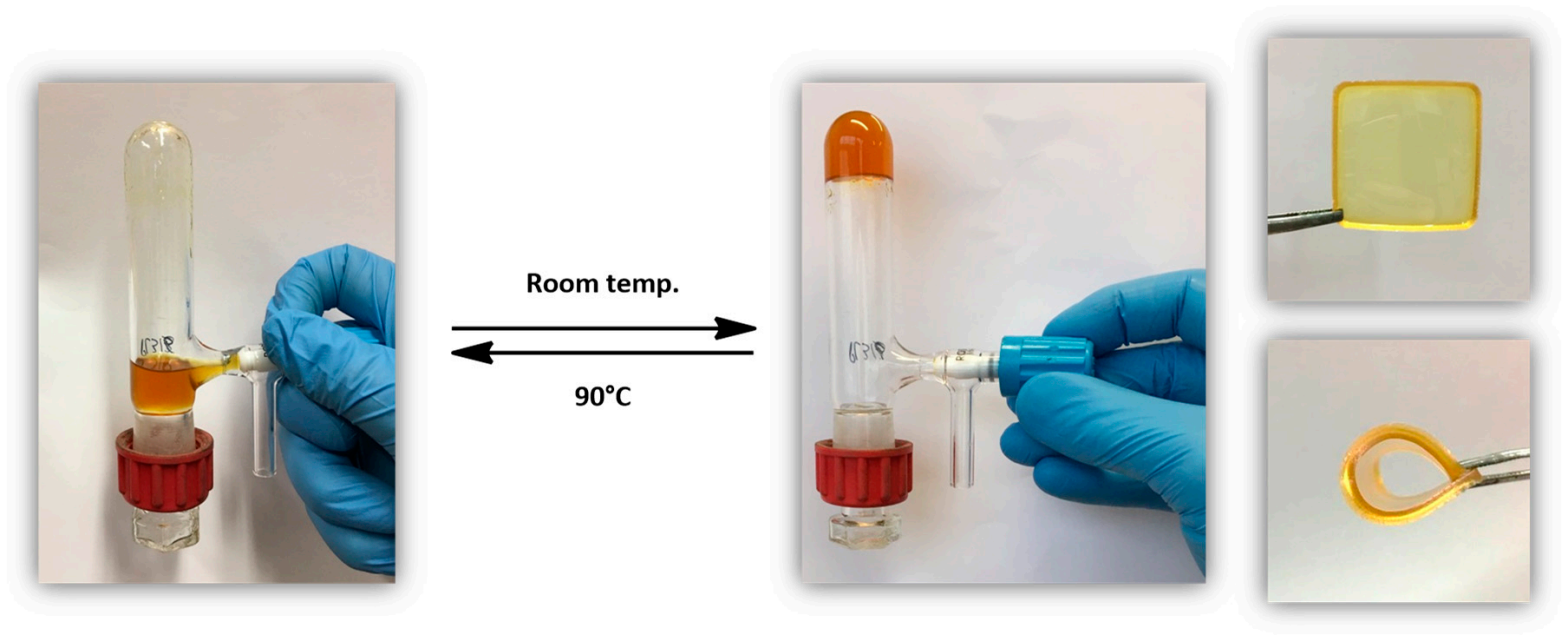
Thermo-reversible bio-based polycarbonate networks: from a soluble polymer solution (**left** picture) to an insoluble network (**right** pictures).

Placed in chloroform, this cross-linked polycarbonate should be back in solution upon heating at 90 °C for 10 min. (retro-Diels–Alder reaction). Figure 3 shows the solubility of the polymer after heating at 90 °C and the formation of an insoluble network when the solution is cooled down to room temperature. These transformations can be done several times in a row without any decomposition of the polymer.

**Figure 4:**
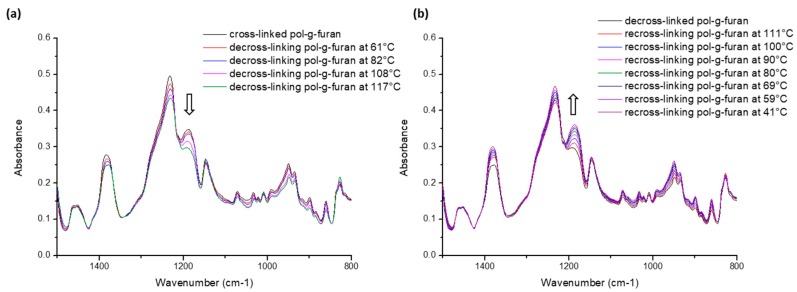
FT-IR traces following (**a**) the de-cross-linking reaction upon heating and (**b**) the cross-linking reaction upon cooling.

FT-IR analysis was performed to demonstrate the reversibility of the cross-linking reaction (Figure 4). First, de-cross-linking of the material was followed by increasing the temperature (2 °C/min) (Figure 4a). The decreasing of the characteristic DA cyclo-adduct peak at 1190 cm^−1^ confirms the de-cross-linking reaction. Once thede-cross-linking of the material was achieved, the temperature was allowed to slowly decrease (2 °C/min). The re-cross-linking reaction was followed by transmission FT-IR (Figure 4b). This time, a significant increase of the DA cyclo-adduct peak was observed at 1190 cm^−1^, highlighting the re-cross-linking reaction.

Figure S4 shows the stacked transmission FT-IR spectra of DA (de/re)-cross-linked furan-containing polycarbonate. Some characteristic furan peaks, such as ν(COC) at 1013 cm^−1^ and ν(C-H for mono-substituted furan) at 710 cm^−1^, decrease upon cross-linking and increase after de-cross-linking. The ν(COC) peak, for example, is clearly visible in the spectrum of f_85_-P(**NH-Und-6CC**). Its magnitude decreases upon the addition of the bis-maleimide cross-linking agent (cross-linked f_85_-P(**NH-Und-6CC**)). Directly after heating the sample at 120 °C, the magnitude of this peak increases again as the furan groups are decoupled (de-cross-linked f_85_-P(**NH-Und-6CC**)). The peak disappears again after the sample has been cooled down at room temperature (re-cross-linked f_85_-P(**NH-Und-6CC**)). The reverse phenomenon is observed with the cyclo-adduct peak ν(DA cycloadduct) at 1190 cm^−1^.

Both of the observations prove cross-linking and decross-linking take place via a reversible DA reaction between the grafted furan groups and the added bis-maleimide cross-linking agents.

**Figure 5:**
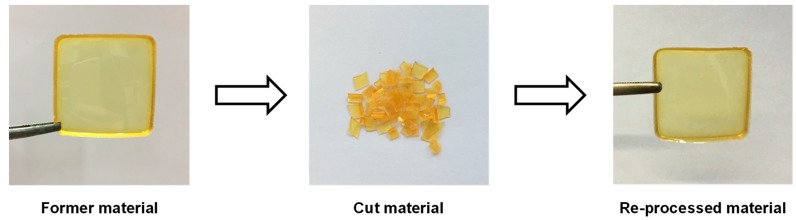
Reprocessability of the cross-linked polycarbonate material.

The film obtained with f_51_-P(**NH-Und-6CC**) was cut in several pieces then re-mold in order to demonstrate further the reprocessability of the cross-linked material, as illustrated in Figure 5. Subsequently, the mechanical properties of the reprocessed materials were compared to the pristine ones to gauge self-healing behaviors.

**Figure 6:**
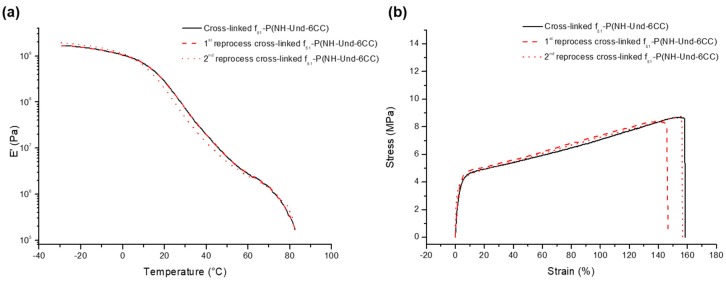
Reprocessability testing: (**a**) dynamic mechanical analyses (DMA) traces and (**b**) Strain-Stress traces of f_51_-P(NH-Und-6CC).

Dynamic Mechanical Analysis (DMA) measured the temperature response of the material’s mechanical behavior (Figure 6a). The moduli of the samples were determined while heating and cooling at a controlled rate (4 °C/min.) with an oscillation frequency of 1 Hz and a strain of 0.04%. The tensile tests at room temperature were also performed with a strain rate of 50 mm/min on three samples (Figure 6b).

The DMA traces show that DA cross-linked polycarbonate, before and after two reprocessing, display exactly the same mechanical behavior upon heating (Figure 6a). Indeed, the storage modulus (E′), which largely determines the elasticity of the rubber, follows the same trend before and after reprocessing. The cross-linked samples show high moduli (>1000 MPa) before 0 °C (glassy state), with only a modest decrease upon heating. A broad glass transition is then observed from 0 °C to 50 °C associated with a significant decrease of E’ upon heating. A drastic loose of mechanical resistance appears after 70 °C, although a very narrow elastic plateau (E’ ≈ 3 MPa) from 50 °C to 70 °C (rubbery state) is detected where the decrease of E’ is less marked, which testifies the start of the retro-DA reaction (de-cross-linking of the material).

Following the same trend, the tensile tests prove that the reprocessing of the polycarbonate materials does not affect its mechanical properties, such as young modulus, elongation at break, and strain at break (Figure 6b).

The next part discusses the influence of the cross-linking density on the mechanical properties of these materials, aving demonstrated the thermo-reversibility of the DA cross-linking and the reprocessing ability of the so-formed cross-linked polycarbonates.

### 2.3. Tuneable Network Properties

In this part, we aim at investigating the influence of several parameters on the thermo-mechanical properties of the networks. For this purpose, the cross-linking procedure exposed in Scheme 2 was applied to all the polycarbonates with different furan contents. The thermo-mechanical properties of the DA cross-linked polycarbonates are summarized in Table 2.

Table 2 shows that the DA cross-linked polycarbonates are not very thermally stable. Indeed, the decomposition of the networks starts at around 110 °C, which is just after the retro-DA reaction temperature. Such a feature can be a limitation for these materials. Table 2 also indicates that the T_g_ of the different cross-linked polycarbonates range from 4.6 °C to 13.4 °C. These values are not following a trend with respect to the furan content grafted on the P(**NH-Und-6CC**). Indeed, the highest furan content network (i.e., 88 mol%) that has potentially the highest cross-linking density, exhibits a T_g_ of 4.6 °C, while the network that was prepared from the linear precursor only containing 31 mol% of furan moieties displays a higher T_g_ of 10.2 °C. At a first glance, this result seems to be not logical, but while taking into account that the T_g_ of the polymer f_88_-P(**NH-Und-6CC**) is −59.8 °C and those of f_31_-P(**NH-Und-6CC**), −6.6 °C, the difference between the T_g_ of the network and the T_g_ of the linear precursor is much more significant for high furan content samples.

Nevertheless, the mechanical properties of the networks evaluated at room temperature will be influenced by the state of the material (rubbery or glassy) and not only by the cross-linking density, due to these different T_g_ values.

**Figure 7:**
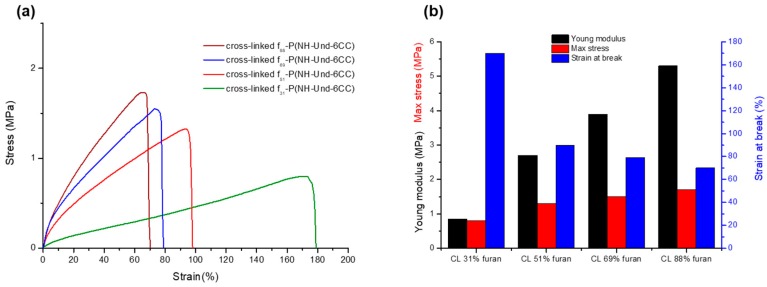
(**a**) Tensile tests performed at 65 °C for various polycarbonate materials and (**b**) the corresponding Young’s modulus, max stress, and strain at break.

Thus, the mechanical properties were tested at 65 °C, where all of the cross-linked polymers are in a rubbery state and before the r-DA. Tensile tests were performed on the various cross-linked samples (Figure 7a). The tensile strength, the Young’s modulus and the elongation at break were determined from these tensile tests and averaged over four measurements (Figure 7b). These values are also reported in Table 2. Figure 7 shows that the high furan-containing materials exhibit significantly higher tensile modulus and lower elongation at break values when compared to those containing lower furan moieties. This is typical, as high tensile moduli and low elongation values are indicative of material with high cross-linking densities. Consequently, the cross-linking density increases as a function of the dangling furan content in the former polycarbonate, as expected.

**Figure 8:**
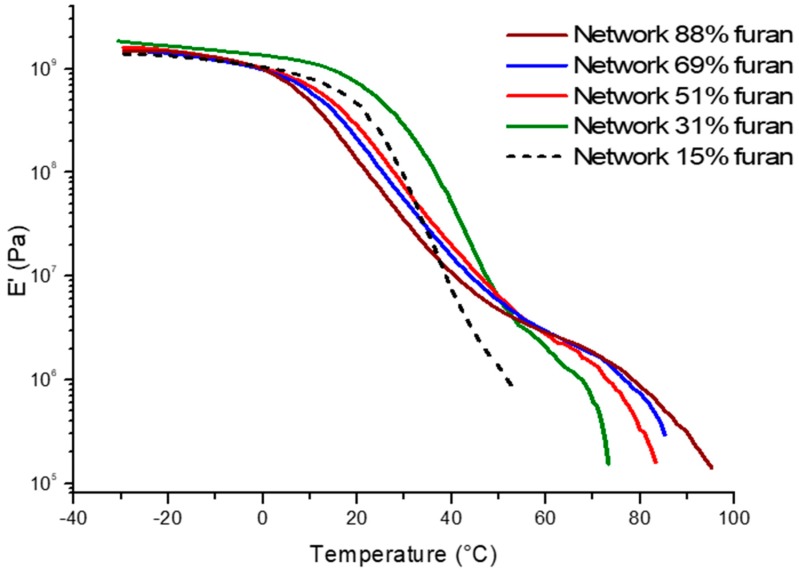
DMA traces for cross-linked polycarbonates samples.

In addition, DMA measured the mechanical behaviour with temperature of the cross-linked polycarbonates (Figure 8). The test was performed under the same conditions than mentioned previously.

Figure 8 confirms the results that were obtained by DSC i.e., the T_α_ slightly decreases with the increase of cross-linking density. Additionally, the cross-linking density also influences the retro DA reaction. Indeed, the DMA traces show that the higher the cross-linking density, the later the retro-DA reaction. The slope of the curve corresponding to the rDA is also less steep, meaning that the rDA reaction is slower.

Finally, gel content and swelling ratio of the cross-linked materials provide information about the cross-linking density. A high gel content combined with a low swelling ratio indicates that the material is highly cross-linked. The gel fraction [33] was calculated according to

(1) $$Gel\;fraction\left(\%\right)=\frac{m_{d}}{m_{0}}\times 100$$

where *m_d_* is the mass of dried (extracted) samples and *m*_0_ is the mass of the specimens before swelling. Equation (1): Formula used to calculate the gel fraction.

The swelling ratio was calculated according to Equation (2), where m_t_ is the mass of the swollen samples in DCM.

(2) $$Swelling\;ratio\;\left(\%\right)=\frac{m_{t}-m_{d}}{m_{d}}\times 100$$

Equation (2): Formula used to calculate the swelling ratio.

Table 2 provides the gel contents and the equilibrium swelling ratios values of the networks. The effect of the pendent furan content on the polymer can be clearly seen. The resulting polycarbonate networks had greatly increased gel contents and decreased swelling ratios with increasing pendent furan rings content. Indeed, the gel content of the network increases from 71% to 96.5% for 31 mol% and 88 mol% of pendent furan content, respectively. Simultaneously, the swelling ratio of the network decreased from 765% to 238% with the above-mentioned pendent furan content. As expected, the cross-linking density is higher when high furan content is grafted on P(**NH-Und-6CC**).

## 3. Materials and Methods

### 3.1. Materials

All of the products and solvents (reagent grade) were used as received, except otherwise mentioned. The solvents were of reagent grade quality and they were purified whenever necessary according to the methods reported in the literature. The polycarbonate, P(NH-Und-6CC) has been synthesized, as previously described [21].

### 3.2. Characterization

^1^H and ^13^C-NMR spectra were recorded on Bruker Avance 400 spectrometer (400.20 MHz or 400.33 MHz and 100.63 MHz for ^1^H and ^13^C, respectively, Bruker, Wissembourg, France) by using CDCl_3_ as a solvent at room temperature, except otherwise mentioned. Two-dimensional analyses, such as ^1^H-^1^H COSY (COrrelation SpectroscopY), and ^1^H-^13^C HSQC (Heteronuclear Single Quantum Spectroscopy) were also performed.

Size Exclusion Chromatography (SEC) analyses were performed in THF (25 °C) on a PL GPC50 with four TSK columns: HXL-L (guard column), G4000HXL (particles of 5 mm, pore size of 200A, and exclusion limit of 400,000 g/mol), G3000HXL (particles of 5 mm, pore size of 75 A, and exclusion limit of 60,000 g/mol), G2000HXL (particles of 5 mm, pore size of 20 A, and exclusion limit of 10,000 g/mol), at an elution rate of 1 mL/min. The elution times of the filtered samples were monitored while using UV and RI detectors and SEC were calibrated using polystyrene standards.

The differential scanning calorimetry (DSC) thermograms were measured using a DSC Q100 apparatus from TA instruments (New Castle, DE, USA). For each sample, two cycles from −80 to 100 °C at 10 °C min^−1^ were performed and then the glass transition and melting temperatures were calculated from the second heating run.

Thermogravimetric (TGA) analyses were performed on TGA-Q50 system from TA instruments (New Castle, DE, USA) at a heating rate of 10 °C min^−1^ under nitrogen atmosphere from room temperature to 600 °C.

The dynamic mechanical analyses (DMA) were performed on RSA 3 (TA instrument, New Castle, DE, USA). The sample temperature was modulated from −50 °C to 150 °C, depending on the sample at a heating rate of 4 °C min^−1^.

Tensile tests were performed on a MTS Qtest 25 Elite controller (Cary, NC, USA) at room temperature. The initial grip separation was set at 20 mm and the crosshead speed at 50 mm min^−1^. The results were obtained from at least three replicates for each sample. Tensile test at 65 °C was also performed on one sample while using the same procedure.

Strip-shaped specimens (0.6 mm thick, 0.5 mm width, 3 mm length) were placed in 10 mL of DCM for 48 h to determine the equilibrium swelling ratios and gel contents. DCM was replaced once after 24 h. We assume that this procedure ensured complete removal of the sol fraction. Subsequently, the swollen gels were dried to constant weight at room temperature in vacuum and weighed.

### 3.3. General Procedure for the Synthesis of Furan-Functionalized fx-P(NH-Und-6CC)

In a round-bottom flask equipped with magnetic stirrer, P(**NH-Und-6CC**) (1 equiv.), furfuryl mercaptan (3 equiv.) and DMPA (1 mol%) were dissolved in ethanol (2 mol/L^−1^). The reaction was allowed to proceed at room temperature during the appropriate period of time under UV irradiation through an optical fiber (365 nm). The solvent was removed to produce f_x_-P(**NH-Und-6CC**), which was purified by precipitation in cold methanol (−63 °C, mixture liquid nitrogen/chloroform). The functional polymer was obtained as waxy solid. Yield = 80–90%, depending on the degree of functionalization.

### 3.4. General Procedure for Polycarbonates Cross-Linking by Diels-Alder Reaction

The polymer f_x_-P(**NH-Und-6CC**) was dissolved in chloroform (CHCl_3_) at 1 g mL^−1^ in a vial. The bis-maleimide cross-linking agent was then added to the previous solution (0.5 equiv. with respect to furan groups) and the mixture was homogenized by vortex stirring until a clear yellow solution appeared. The vial was closed tightly and then heated up to 60 °C for 10 min.

The warm solution was then poured in a Teflon mold, and the chloroform was allowed to gently evaporate overnight at room temperature. A cross-linked film CL-f_x_-P(**NH-Und-6CC**) was obtained and then dried under reduced pressure for several hours to remove traces of chloroform. DSC, TGA, DMA, and tensile tests characterized the obtained cross-linked film.

### 3.5. General Procedure for Polycarbonates Decross-Linking by Retro-Diels–Alder Reaction

Placed in chloroform, the cross-linked polycarbonate CL-f_x_-P(**NH-Und-6CC**) was heated at 90 °C for 10 min. The retro-DA reaction occurs and the polymer is dissolved in the chloroform. These transformations can be done several times in a row without any decomposition of the polymer.

## 4. Conclusions

To conclude, several furan-containing polycarbonates were synthesized via the thiol-ene reaction between P(**NH-Und-6CC**) and furfuryl mercaptan. The content of dangling furan rings that were grafted on the polymer backbone can be tuned by monitoring the reaction time. Such furan-functionalized polycarbonates were then cross-linked thanks to the Diels–Alder reaction with a bis-maleimide as cross-linking agent. The reversibility of the cross-linking reaction was proven by FT-IR analyses. Moreover, cross-linked polycarbonate materials have been reprocessed twice without altering their mechanical properties. Finally, the thermo-reversible DA reaction affords the preparation of reversible cross-linked polycarbonate materials with tuneable properties as a function of the pendent furan content that was grafted on the polycarbonate backbone.

While, the poor thermal stability of the furan-functionalized cross-linked polycarbonates represent a hindrance for other applications, the possibility to decrosslink the network around 70 °C could be an advantage in biomedical applications.

## Figures and Tables

**Table 1:** Characteristics of f_x_-P(NH-Und-6CC) with pendent furan moieties.

Polymer	Reaction Time (h)	Furan Content ^a^ (mol%)	M_n_^b^ (g mol^−1^)	[Ɖ] ^b^	T_g_ (°C) ^c^
P(NH-Und-6CC)	0	0	5700	1.07	23.1
f_31_-P(NH-Und-6CC)	1	31	6200	1.15	−6.6
f_51_-P(NH-Und-6CC)	2	51	6650	1.12	−13.5
f_69_-P(NH-Und-6CC)	4	69	7300	1.19	−49.0
f_88_-P(NH-Und-6CC)	12	88	7850	1.22	−59.6

^a^: Determined by ^1^H NMR; ^b^: Determined by SEC in THF (PS Std); ^c^: Determined by DSC at 10 °C min^−1^ from the second cycle (see Figure S3).

**Table 2:** Thermo-mechanical properties of Diels–Alder (DA) cross-linked polycarbonate materials.

			Performed at RT	Performed at 65 °C				
Furan Content ^a^	T_d(5%)_ f_x_-Network (°C) ^b^	T_g_ f_x_-Network (°C) ^c^	Young Modulus (MPa) ^d^	Young Modulus (MPa) ^e^	Max Stress (MPa) ^e^	Elongation at Break (%) ^e^	Gel Content (%)	Swelling Ratio (%)
88%	108	4.6	31 ± 2	5.3	1.7	70	96.5	238
69%	107	6.8	43 ± 2	3.9	1.5	79	93.7	296
51%	110	13.4	90 ± 15	2.7	1.3	98	96	318
31%	112	10.2	230 ± 70	0.85	0.8	170	71	765

^a^ Calculated with ^1^H NMR. ^b^ Measured by TGA analysis. ^c^ Measured by DSC analysis (see Figure S5). ^d^ Calculated with tensile tests at RT. ^e^ Calculated with tensile tests at 65 °C.

## Supplementary Materials

The following are available online at https://www.mdpi.com/1420-3049/25/1/74/s1, Experimental procedure to synthesize P(NH-Und-6CC), Figure S1: Stacked ^1^H NMR monitoring the reaction between furfuryl mercaptan and P(NH-Und-6CC); Figure S2: SEC traces in THF of fx-P(NH-Und-6CC) as a function of furan content; Figure S3: DSC traces of furan-functionalized P(NH-Und-6CC); Figure S4: FT-IR absorption spectra of furan-containing polycarbonate and DA (de/re)-cross-linked furan-containing polycarbonate; Figure S5: DSC traces of cross-linked furan-functionalized P(NH-Und-6CC); Figure S6 TGA traces of cross-linked furan-functionalized P(NH-Und-6CC).
