# Supplementary material for: Bio-Based Thermo-Reversible Aliphatic Polycarbonate Network
Source: Molecules. 2019 Dec 24;25(1):74. doi: 10.3390/molecules25010074 (PMC6982953; doi:10.3390/molecules25010074)
Supplement: Supplementary file 1 [file molecules-25-00074-s001.pdf]

Bio-based thermo-reversible aliphatic polycarbonate network

Pierre-Luc Durand [1], Etienne Grau [1] and Henri Cramail [1*]

[1] Univ. Bordeaux, CNRS, Bordeaux INP, LCPO, UMR 5629, F-33600, Pessac, France
* Correspondence: cramail@enscbp.fr (H.C.)

Synthesis of **NH-Und-1,3-diol**

Methyl 10-undecenoate (1 equiv.), was mixed with 2-amino-1,3-propanediol (1.3 equiv.). TBD (0.05 equiv.) was added to the reaction mixture and heated up at 80°C under a nitrogen flow to remove methanol formed. The reaction mixture was heated for 3h. Depending on the matrix, the reaction mixture might become solid indicating the end of the reaction. The product is then extracted with $CHCl_3$ and washed 3 times with water. Brine is added in case of emulsion. The organic phase was dried over Na_2SO_4 and the solvent was removed under vacuum to yield **NH-Und-1,3-diol**. The diol was obtained as white crystals. Yield: 70%. ^{1}H NMR (400 MHz, $CDCl_3$) δ (ppm): 6.22 (s, NH), 5.81 (m, 1H), 4.93 (m, 2H), 3.96 (m, 1H), 3.84 (m, 2H), 3.78 (m, 2H), 2.72 (2 OH), 2.24 (t, 2H), 2.04 (q, 2H), 1.65 (m, 2H), 1.38-1.25 (m, 10H). ^{13}C NMR (100 MHz, $CDCl_3$) δ (ppm): 174.39 (CH_2-CO-NH), 139.33 (CH_2=CH-CH_2), 114.31 (CH_2=CH-CH_2), 63.76 (CH-(CH_2-OH)$_2$), 52.49 (CH-(CH_2-OH)$_2$), 36.94 (CH_2-CO-NH), 33.92 (CH_2=CH-CH_2), 29.44-29.02 (CH_2), 25.84 (CH_2-CH_2-CO-NH).

Synthesis of **NH-Und-6CC**

In a 500 mL round bottom flask at 0°C equipped with magnetic stirrer, **NH-Und-1,3-diol** (1.0 equiv.) and ethyl chloroformate (4.0 equiv.) were dissolved in 300 mL THF. To the cold reaction mixture, triethylamine (4.0 equiv.) was added dropwise over 10 min. The reaction was allowed to proceed in ice-cold conditions for about 1 hour and then allowed to proceed at room temperature overnight. The precipitated solids were filtered off and the volatiles were removed to result in crude product, which was subjected to flash column chromatography, using a gradient of DCM (100 %) to DCM (95 %) and methanol (5 %) solvent mixture, followed by the removal of volatiles to result in white solid **NH-Und-6CC** as the functional monomer. Yield: 55%. ^{1}H NMR (400 MHz, $CDCl_3$) δ (ppm): 7.63 (s, NH), 5.76 (m, 1H), 4.93 (m, 2H), 4.54-4.40 (m, 5H), 2.22 (t, 2H), 1.99 (q, 2H), 1.60 (t, 2H), 1.25 (m, 10H). ^{13}C NMR (100 MHz, $CDCl_3$) δ (ppm): 174.33 (CH_2-CO-NH), 148.66 (OCOO), 139.19 (CH_2=CH-CH_2), 114.21 (CH_2=CH-CH_2), 71.45 (CH-(CH_2-OCOO)$_2$), 40.94 (CH-(CH_2-OCOO)$_2$), 36.14 (CH_2-CO-NH), 33.81 (CH_2=CH-CH_2), 29.34-28.93 (CH_2), 25.57 (CH_2-CH_2-CO-NH).

Polymerization of **NH-Und-6CC**

All polymerizations were performed under inert atmosphere (nitrogen) using standard Schlenk, vacuum line, and glovebox techniques. In a 15 mL schlenk flask containing a magnetic stirrer, in glove box, **NH-Und-6CC** (50 equiv.), BnOH (2.0 equiv.) and Schreiner TU (1.0 equiv.) were dissolved in dry DCM ([**NH-Und-6CC**] = 2 mol.L^{-1}). To this solution, DBU (1.0 equiv.) was added to initiate polymerization. The reaction mixture was allowed to stir at room temperature. After 5h, the reaction was quenched by the addition of benzoic acid (2 equiv.) and purified by precipitation in cold methanol to yield P(**NH-Und-6CC**). The polymer was obtained as white solid. Yield: 91%. ^{1}H NMR (400 MHz, $CDCl_3$) δ (ppm): 5.80 (m, 1H), 4.93 (d, 2H), 4.64-3.93 (m, 5H), 2.24 (m, 2H), 2.01 (q, 2H), 1.62 (m, 2H), 1.28 (m, 10H). SEC (THF, RI): Mn= 5 700 g.mol^{-1}, Đ=1.07.

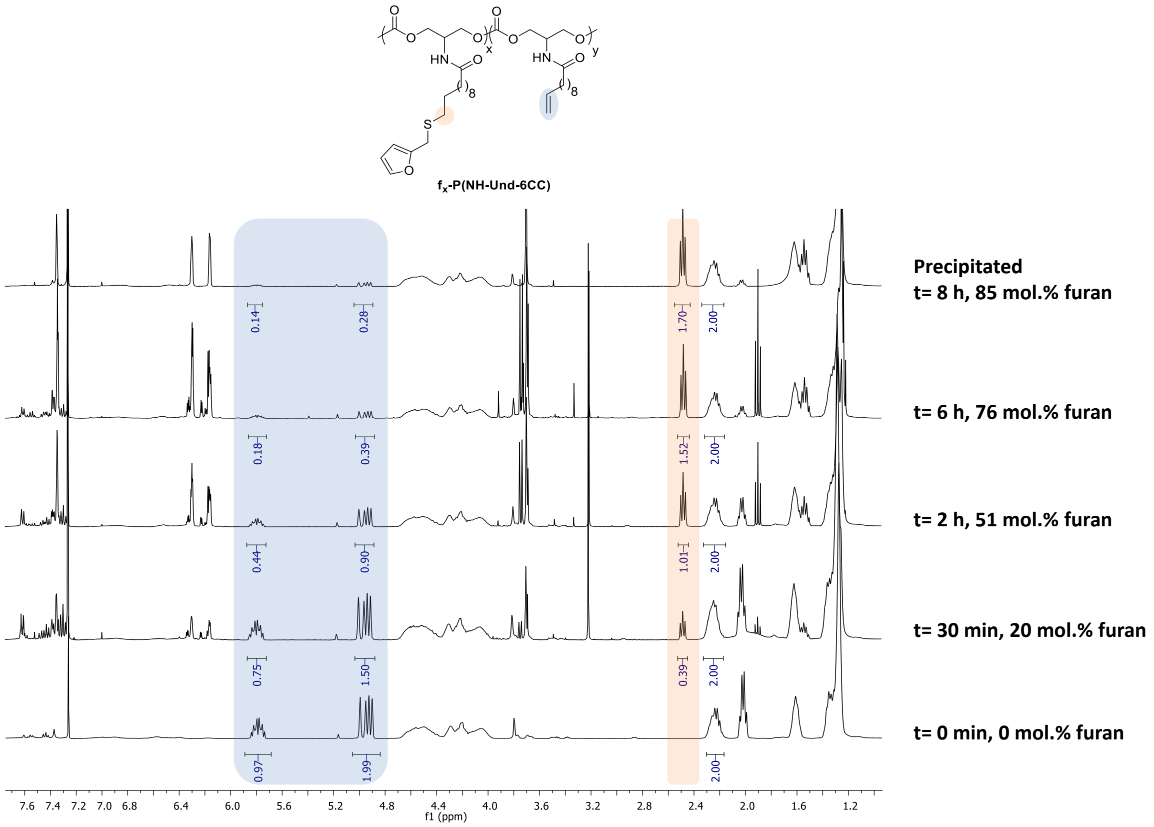

Figure S1: Stacked ^{1}H NMR monitoring the reaction between furfuryl mercaptan and P(NH-Und-6CC)

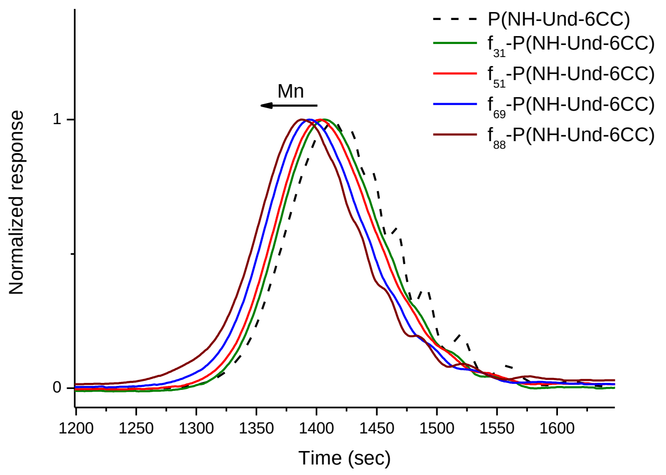

Figure S2: SEC traces in THF of f_x-P(NH-Und-6CC) as a function of furan content

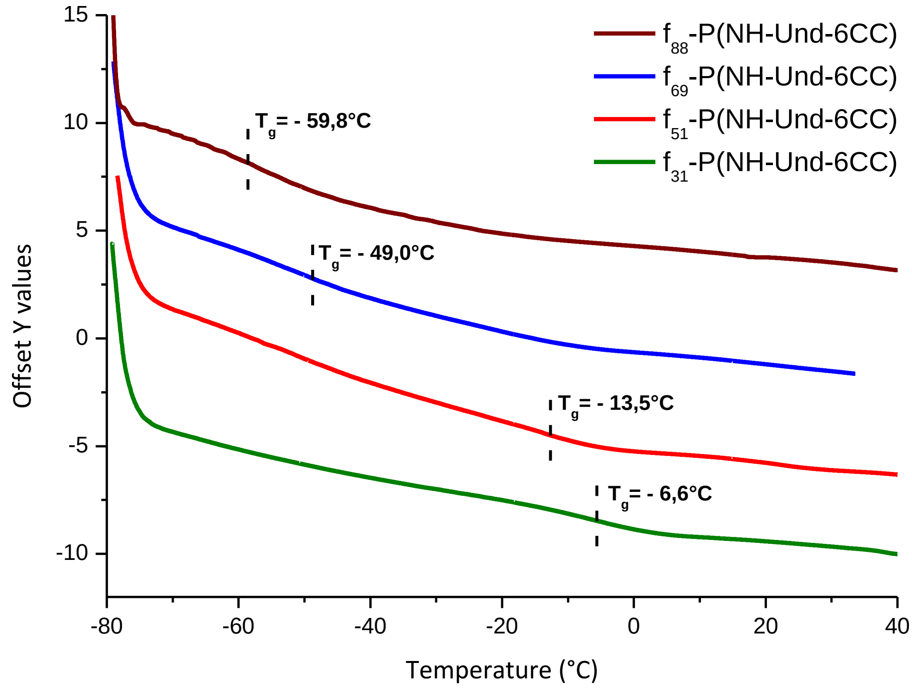

Figure S3: DSC traces of furan-functionalized P(NH-Und-6CC)

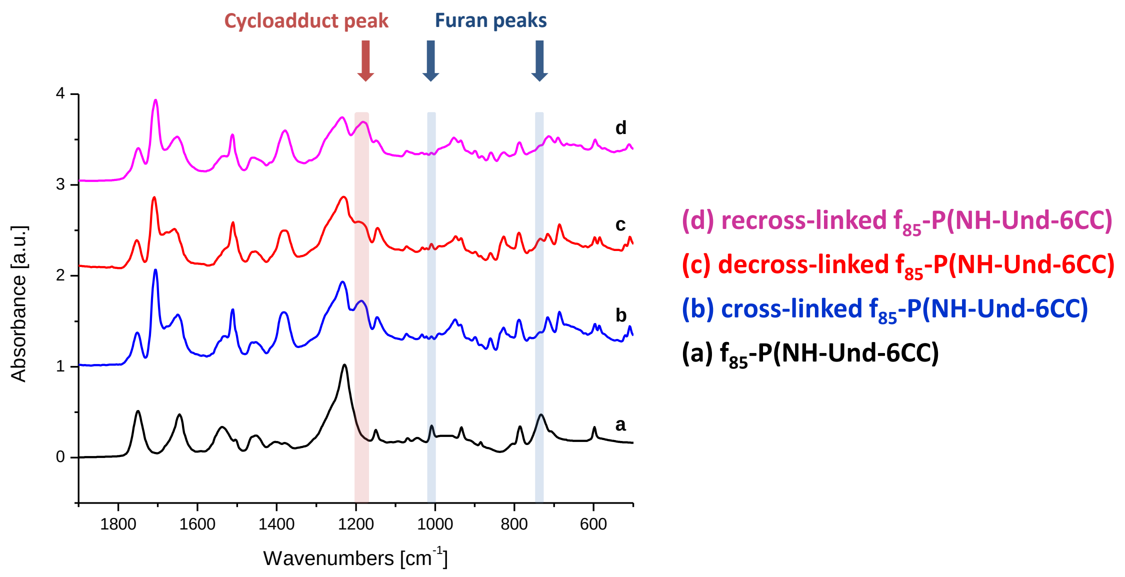

Figure S4: FT-IR absorption spectra of furan-containing polycarbonate and DA (de/re)-cross-linked furan-containing polycarbonate

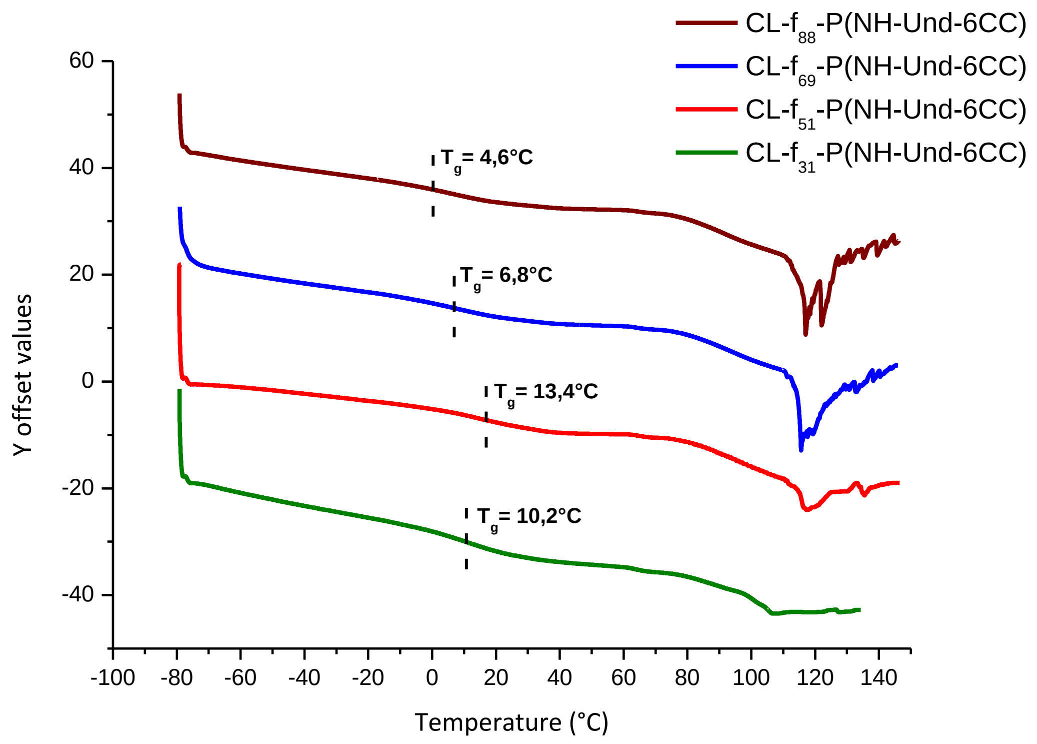

Figure S5: DSC traces of cross-linked furan-functionalized P(NH-Und-6CC)

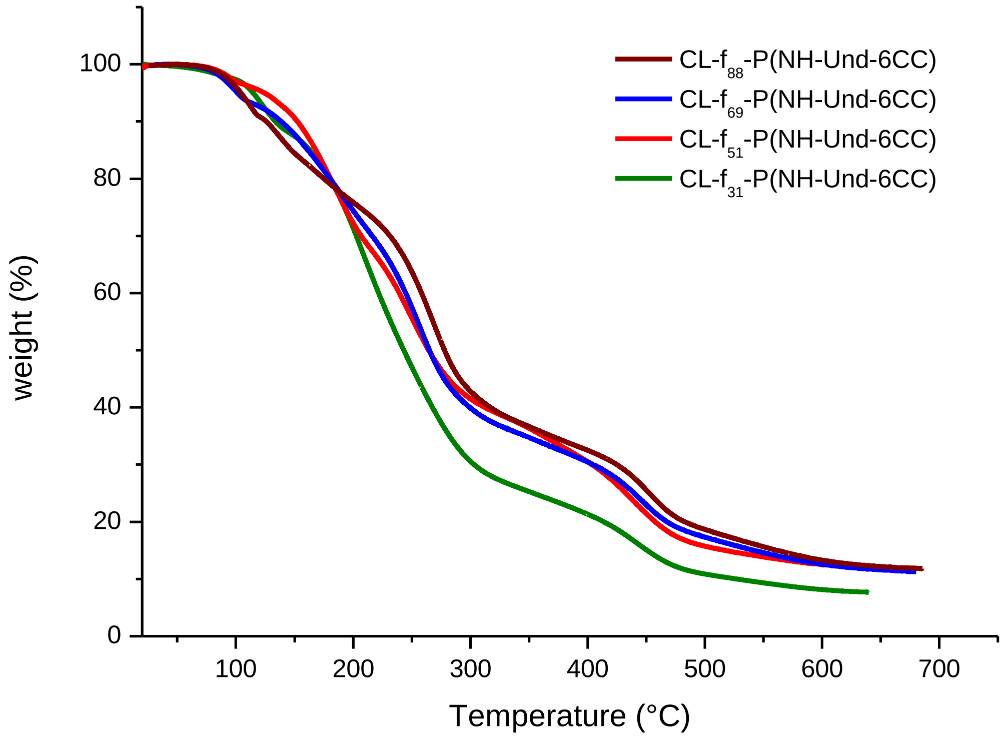

Figure S6: TGA traces of cross-linked furan-functionalized P(NH-Und-6CC)
